# Efficacy and safety of modified Xuanbai Chengqi Decoction as an adjunctive treatment for severe pneumonia: a systematic review and meta-analysis

**DOI:** 10.3389/fphar.2025.1573025

**Published:** 2025-04-24

**Authors:** Yifu Tie, Han Liu, Tong Zhang, Tianwei Meng, Qun Liang

**Affiliations:** ^1^ Heilongjiang University of Chinese Medicine, Harbin, China; ^2^ Institute for Global Health, University College London, London, United Kingdom; ^3^ Critical Care Medicine, The First Affiliated Hospital, Heilongjiang University of Chinese Medicine, Harbin, China

**Keywords:** severe pneumonia, pneumonia, meta-analysis, traditional Chinese medicine, symptom complex

## Abstract

**Objective:**

To evaluate the efficacy and safety of Modified Xuanbai Chengqi Decoction as an adjunctive therapy for severe pneumonia (SP) and to explore its clinical rationale.

**Methods:**

A comprehensive search was performed in CNKI, Wanfang, VIP, CBM, Cochrane Library, PubMed, and Embase, covering database inception to November 2024. Randomized controlled trials that investigated the combination of Modified Xuanbai Chengqi Decoction with standard therapy for severe pneumonia were included. Quality evaluation, meta-analysis, and bias assessments were performed with Review Manager 5.4, using risk ratios and mean differences (MD), both with 95% confidence intervals to estimate effects.

**Results:**

A total of 14 RCTs involving 1,061 participants met the inclusion criteria. Meta-analysis indicated that adjunctive treatment with the decoction achieved better overall response rates [RR = 1.21, 95% CI (1.14, 1.28), P < 0.00001] and demonstrated significant reductions in interleukin-6 (IL-6, MD = −15.07, 95% CI (−17.31, −12.83), P < 0.00001), interleukin-13 (IL-13, MD = −7.30, 95% CI (−7.82, −6.79), P < 0.00001), and tumour necrosis factor-α (TNF-α, MD = −0.56, 95% CI (−0.64, −0.48), P < 0.00001), high-sensitivity C-reactive protein (hs-CRP, MD = −2.09, 95% CI (−2.47, −1.71), P < 0.00001), procalcitonin (PCT, MD = −2.04, 95% CI (−2.55, −1.53), P < 0.00001), arterial blood gas lactate (LaC, MD = −2.28, 95% CI (−2.45, −2.11), P < 0.00001), mechanical ventilation time (MD = −120.03, 95% CI (−130.14, −109.92), P < 0.00001), clinical pulmonary infection score (CPIS, MD = −2.71, 95% CI (−3.95, −1.82), P < 0.00001), Acute Physiology and Chronic Health Evaluation II (APACHE II) score (MD = −7.41, 95% CI (−7.54, −7.28), P < 0.00001), and ICU transfer rate [RR = 22.88, 95% CI (6.68, 78.32), P < 0.00001].

**Conclusion:**

Modified Xuanbai Chengqi Decoction appears advantageous as an adjunct for SP treatment, but the evidence remains insufficient to support widespread application owing to the low quality of the included research. Future studies should incorporate higher-quality RCTs and assess drug safety and cost-effectiveness in a rigorous manner.

## 1 Introduction

Severe pneumonia (SP)continues to pose a substantial global health burden (Haessler et al., 2022), It is characterized by acute inflammatory changes in the lung parenchyma due to infections caused by various pathogens, such as bacteria, viruses, or fungi. According to current research, pneumonia remains one of the leading causes of morbidity and mortality worldwide, especially in vulnerable groups, including older adults, children, and individuals with underlying health conditions (Liu et al., 2023; Yang et al., 2024). The pathophysiological mechanisms are driven by a complex interplay between the host immune system and microbial virulence factors, resulting in dysregulated inflammation, alveolar damage, and impaired gas exchange (Mizgerd, 2017; Li et al., 2024). Clinical manifestations range from mild respiratory distress to life-threatening respiratory failure, requiring timely recognition and management. Recent progress in antibiotic therapy, adjunctive corticosteroids, and supportive measures has improved treatment outcomes to some extent (Nair and Niederman, 2021; Decker et al., 2022; Bergmann et al., 2024). Nonetheless, challenges in managing severe pneumonia persist due to antibiotic resistance (Shorr and Zilberberg, 2021) and the need for individualized therapeutic approaches. Increasing evidence suggests that the lung microbiome plays a vital role in modulating disease severity and recovery, given that interactions between microbe populations and the host immune system can shape clinical outcomes (Li et al., 2023). Traditional Chinese medicine (TCM) therapies such as Xuanbai Chengqi Decoction are gaining attention as potential supportive strategies to control excessive inflammation, although there is a lack of comprehensive systematic evaluations. In light of this, a systematic review and meta-analysis was conducted to assess the clinical efficacy of Xuanbai Chengqi Decoction in managing severe pneumonia.

## 2 Materials and methods

### 2.1 Pharmaceutical ingredient

The combination of Modified Xuanbai Chengqi Decoction (Composition: *Gypsum, Rhubarb, Bitter Apricot Seed, Snakegourd Peel, Immature Bitter Orange, Perilla Seed, Descurainia Seed, Thunberg Fritillary Bulb, Stemona Root, Loquat Leaf, Red Tangerine Peel*, Table 1). Xuanbai Chengqi Decoction, originating from the classical Chinese medical text *Systematic Differentiation of Warm Diseases* (Wen Bing Tiao Bian), was investigated in this study. The original formula consists of *Gypsum, Rhubarb, Bitter Apricot Seed, Snakegourd Peel*. The modified formula used in all trials incorporated seven additional herbal components: *Immature Bitter Orange, Perilla Seed, Descurainia Seed, Thunberg Fritillary Bulb, Stemona Root, Loquat Leaf, Red Tangerine Peel*. All studies adhered to identical preparation protocols; herbal components were decocted in water for 30 min to obtain 400 mL of decoction. Interventions across trials employed a unified standard formula, with explicitly reported dosage and preparation methodology. Formula composition details were comprehensively documented in all included trials.

**TABLE 1:** Detailed table of drug composition.

Drug name	Dosage(g)	Latin name	Family name	Medicinal part	Preparation method
Gypsum	15	*Gypsum Fibrosum*	-	Mineral Medicine	The herbal preparation was decocted according to the prescribed dosage (in grams) for 30 min, strained, and 400 mL of decoction was collected
Rhubarb	9	*Rheum palmatum* L	Polygonaceae	*Rhei Radix et Rhizoma* (Root and Rhizome)	
Immature Bitter Orange	9	*Citrus aurantium* L	Rutaceae	*Aurantii Fructus Immaturus* (Immature Fruit)	
Bitter Apricot Seed	6	*Prunus armeniaca* L	Rosaceae	*Armeniacae Semen Amarum* (Seed)	
Snakegourd Peel	5	*Trichosanthes kirilowii* Maxim	Cucurbitaceae	*Trichosanthis Pericarpium* (Fruit Peel)	
Perilla Seed	15	*Perilla frutescens* (L.) Britt	Lamiaceae	*Perillae Fructus* (Fruit)	
Descurainia Seed	15	*Lepidium apetalum* Willd	Brassicaceae	*Lepidii/Descurainiae Semen* (Seed)	
Thunberg Fritillary Bulb	10	*Fritillaria thunbergii* Miq	Liliaceae	*Fritillariae Thunbergii Bulbus* (Bulb)	
Stemona Root	15	*Stemona japonica* (Blume) Miq	Stemonaceae	*Stemonae Radix* (Root)	
Loquat Leaf	15	*Eriobotrya japonica* (Thunb.) Lindl	Rosaceae	*Eriobotryae Folium* (Leaf)	
Red Tangerine Peel	10	*Citrus reticulata* Blanco	Rutaceae	*Citri Reticulatae Pericarpium Rubrum* (Ripe Pericarp)	

### 2.2 Protocol registration

This systematic review was registered in PROSPERO(CRD42024609199).

### 2.3 Search strategy

Searches were performed in both Chinese (CNKI, Wanfang Digital Periodicals, VIP, SinoMed) and international (Embase, Web of Science, PubMed, and Cochrane Library) databases from inception to November 2024. Chinese search terms included “Xuanbai Chengqi,” “Modified Xuanbai Chengqi Decoction,” “severe pneumonia,” and “pneumonia.” English search terms included “Xuanbai Chengqi,” “Severe pneumonia,” and “Necrotizing Pneumonias,” combined as subject terms and free terms. For instance, the CNKI advanced search combined “Xuanbai Chengqi” with “severe pneumonia,” and a sample PubMed strategy is detailed in Supplementary Table S1.

### 2.4 Inclusion criteria

#### 2.4.1 Study types

Randomized controlled trials (RCTs) in any language were included, regardless of whether a blinding method was used.

#### 2.4.2 Participants

Patients were adults (≥18 years) with severe pneumonia, diagnosed according to recognized criteria, with no limitations on disease duration, sex, or age.

#### 2.4.3 Interventions

The intervention group received Modified Xuanbai Chengqi Decoction alongside standard biomedicine therapy, which included general care, antibiotics, glucocorticoids, mechanical ventilation, and symptomatic treatments. The control group received the same standard Western therapies without the TCM decoction.

#### 2.4.4 Outcomes

Primary outcome was overall response rate; secondary outcomes included IL-6, IL-13, TNF-α, hs-CRP, PCT, LaC, duration of mechanical ventilation, CPIS, APACHE II score, ICU transfer rate, and adverse events.

### 2.5 Exclusion criteria

① duplicate publications; ② studies using other Traditional Chinese Medicine (TCM) treatments in the experimental group besides Modified Xuanbai Chengqi Decoction; ③ studies with incomplete data or where the full text could not be obtained; ④ studies involving patients with additional complications; ⑤ low-quality journal publications; ⑥ review articles, animal studies, and conference abstracts.

### 2.6 Literature screening

After deduplication in NoteExpress, two researchers screened all articles independently for relevance, extracted data, and conducted quality evaluations. Any disagreements were resolved through consensus with a third researcher. Extracted information included authors, publication details, participant characteristics, sample size, interventions, treatment duration, outcomes, and average events.

### 2.7 Quality assessment of studies

The Cochrane Reviewers’ Handbook 6.1.0 risk of bias instrument was employed to evaluate quality, categorizing studies as “low risk,” “high risk,” or “unclear risk.”

### 2.8 Statistical analysis

Data were analyzed with RevMan 5.2. For dichotomous outcomes, RR was used; for continuous outcomes, MD was used. The I^2^ statistic and χ^2^ test assessed inter-study heterogeneity. A fixed-effect model was adopted if I^2^ < 50% and P > 0.1; otherwise, a random-effects model was applied. Subgroup analysis based on different outcome levels and sensitivity analyses were carried out to examine the effect of individual studies on pooled results.

## 3 Results

### 3.1 Literature search

**FIGURE 1:**
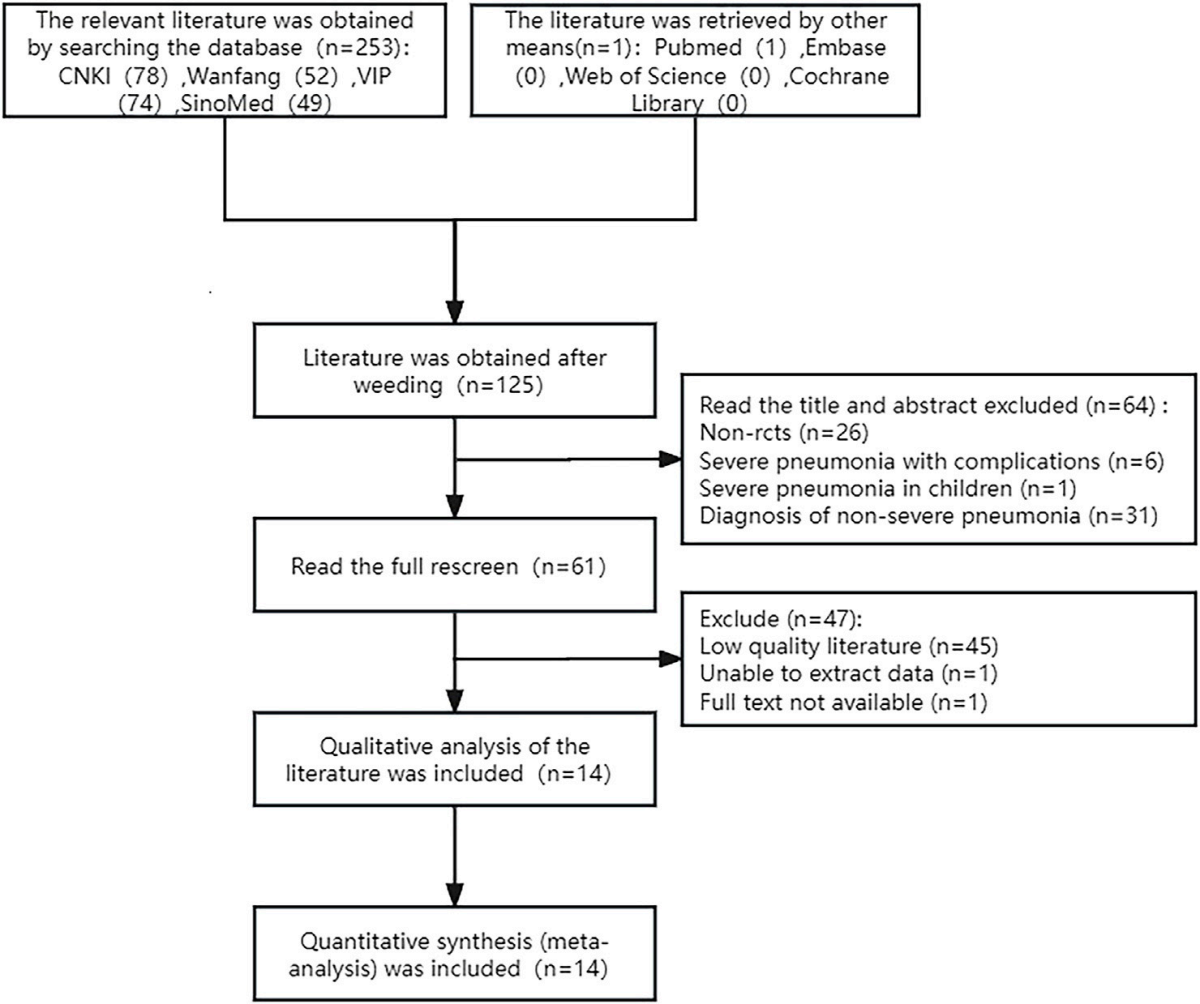
Literature search.

A preliminary search yielded 253 Chinese articles and 1 English article, for a total of 254. After applying the screening criteria, 14 RCTs conducted in China were included. The flow chart of study selection is shown in Figure 1.

### 3.2 Basic information and quality assessment of included studies

**FIGURE 2:**
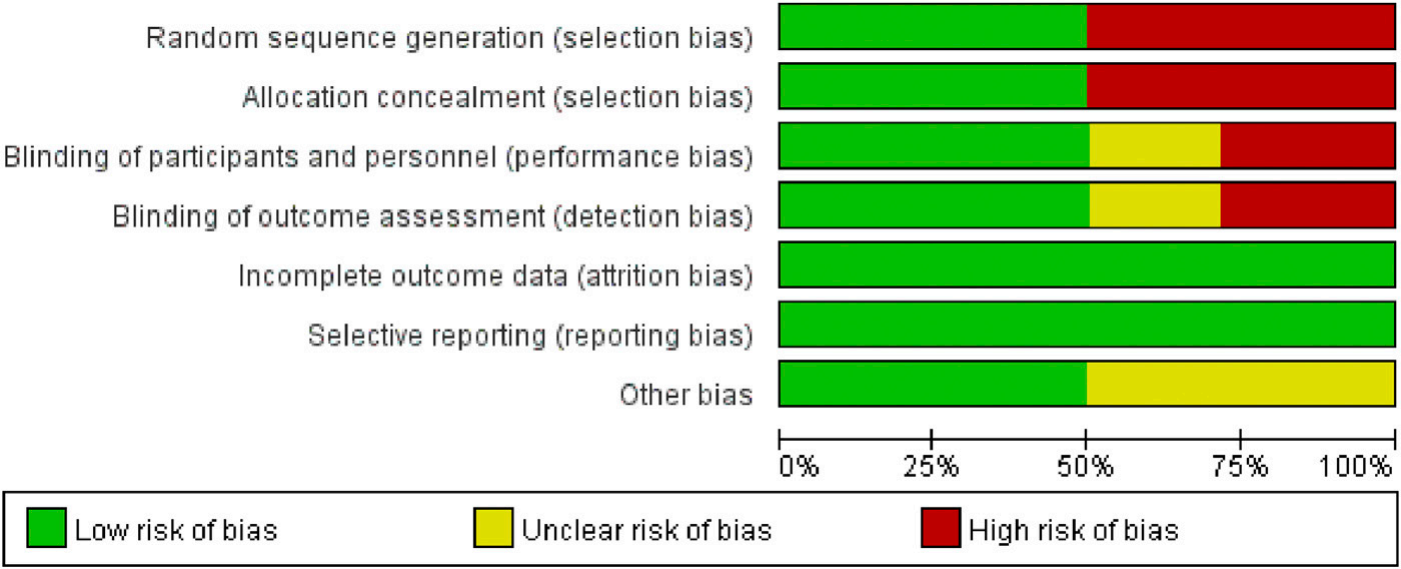
Literature quality assessment.

**FIGURE 3:**
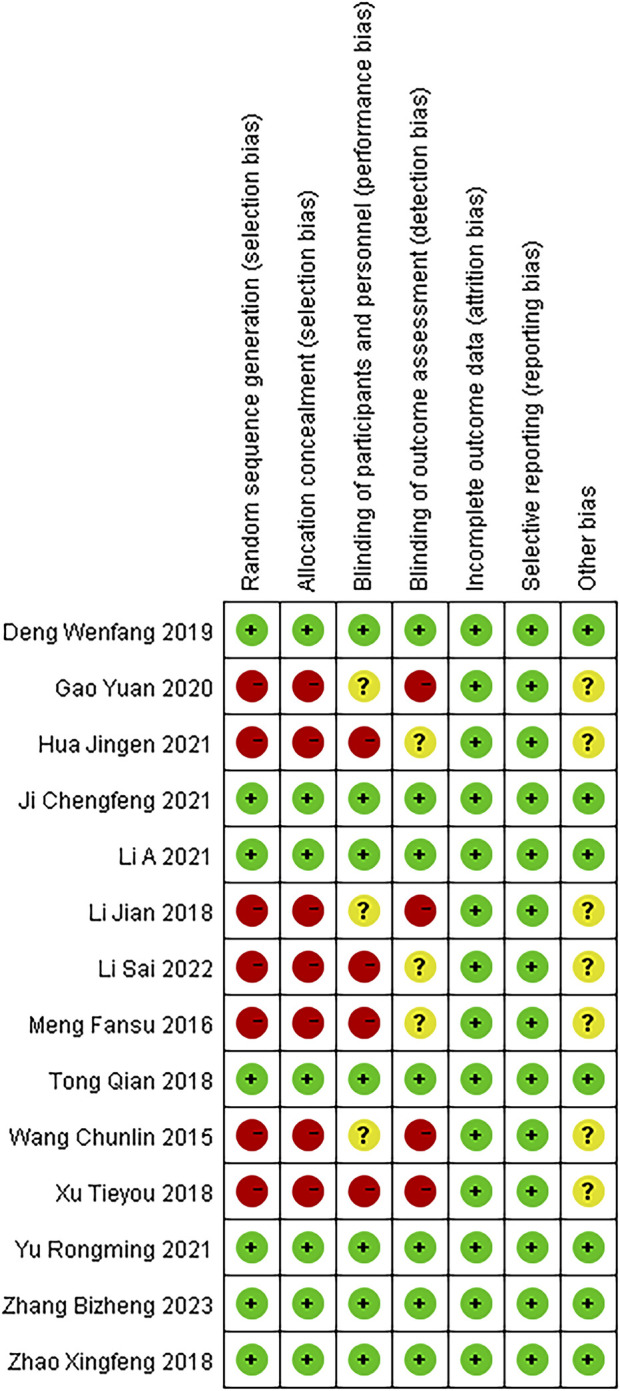
Literature quality assessment.

These 14 RCTs involved 1,061 participants diagnosed with SP (530 in the experimental group and 529 in the control group). All studies stated “randomization,” (Chun-lin wang, 2015; Meng, 2016; Li and Xue 2018; Tong, 2018; Xu T Y et al., 2018; Zhao, 2018; Deng, 2019; Gao, 2020; Hua, 2021; Chengfeng and Ji, 2021; Li et al., 2021; Yu and Hu, 2021; Li S et al., 2022; Zhang et al., 2023) with one specifying a lottery method (Deng, 2019), four citing a random number table (Tong, 2018; Zhao, 2018; Chengfeng and Ji, 2021; Li E et al., 2021), one using simple randomization (Li and Xue, 2018), one employing parallel randomization (Zhang et al., 2023), and seven not reporting the randomization approach (Chun-lin wang, 2015; Meng, 2016; Xu et al., 2018; Gao, 2020; Hua, 2021; Yu and Hu, 2021; Li et al., 2022). All trials compared standard therapy alone with standard therapy plus Modified Xuanbai Chengqi Decoction, and none elaborated on selective reporting or other bias sources. Table 2 summarizes the characteristics, while Figures 2, 3 present the quality evaluation results.

**TABLE 2:** Characteristics of included RCTs (E: experimental group; C: control group).

Author	Year	Country	Sex (M/F)		Age		Syndrome type	Duration (days)	Sample size(n)		Outcome index	Drug usage	Adverse reaction	Modification details
			E	C	E	C			E	C				
Deng Wenfang	2019	China	10/20	17/13	50.2 ± 12.77	50.3 ± 12.76	Phlegm-heat obstructing lung	7 days	30	30	①②⑤⑧	Oral administration	Not mentioned	All studies have added Immature Bitter Orange, Perilla Seed, Descurainia Seed, Thunberg Fritillary Bulb, Stemona Root, Loquat Leaf, Red Tangerine Peel
Gao Yuan	2020	China	11/18	12/17	51.5 ± 10.81	52.3 ± 10.49	Phlegm-heat obstructing lung	7 days	29	29	①②⑤⑥⑦⑧	Oral administration	Not mentioned	
Hua Jingen	2021	China	20/14	18/16	48.43 ± 4.62	49.03 ± 4.45	Phlegm-heat obstructing lung	10 days	34	34	④⑥⑦	Oral administration	Not mentioned	
Ji Chengfeng	2021	China	15/14	13/16	75.23 ± 8.29	76.24 ± 7.33	Phlegm-heat obstructing lung	Nothing	29	29	①②④	Oral administration	Not mentioned	
Li A	2021	China	16/14	11/19	54.9 ± 14.55	55.2 ± 14.25	Phlegm-heat obstructing lung	7 days	30	30	③④⑦	Oral administration	Not mentioned	
Li Jian	2018	China	24/16	23/17	42.1 ± 17.13	42.3 ± 17.42	Phlegm-heat obstructing lung	10 days	40	40	①⑥⑦⑨⑩⑪	Oral administration	Not mentioned	
Li Sai	2022	China	15/15	13/17	52.4 ± 2.0	53.4 ± 2.1	Phlegm-heat obstructing lung	14 days	30	30	①	Oral administration	Not mentioned	
Meng Fansu	2016	China	12/28	24/16	41.9 ± 18.1	42.5 ± 17.9	Phlegm-heat obstructing lung	10 days	40	40	①⑥⑦⑨⑩⑪	Oral administration	Nothing	
Tong Qian	2018	China	31/21	33/19	65.24 ± 2.70	65.13 ± 2.24	Phlegm-heat obstructing lung	10 days	52	52	①⑥⑦⑨⑩	Oral administration	Not mentioned	
Wang Chunlin	2015	China	7/14	6/14	55.0 ± 21.0	53.0 ± 20.0	Not mentioned	7 days	21	20	⑩	Nasal feeding	Not mentioned	
Xu Tieyou	2018	China	23/13	11/25	41.9 ± 18.24	42.3 ± 17.43	Phlegm-heat obstructing lung	10 days	36	36	①⑥⑦⑨	Oral administration	Not mentioned	
Yu Rongming	2021	China	25/15	23/17	58.43 ± 8.05	58.05 ± 8.22	Not mentioned	10 days	40	40	①②⑤⑥⑦	Oral administration	Not mentioned	
Zhang Bizheng	2023	China	12/18	17/13	47.7 ± 2.8	46.9 ± 2.5	Phlegm-heat obstructing lung	7 days	30	30	①②③⑤	Oral administration or Nasal feeding	Not mentioned	
Zhao Xingfeng	2018	China	27/19	25/21	54.18 ± 4.27	54.31 ± 4.29	Phlegm-heat obstructing lung	7 days	46	46	①②⑤⑧	Oral administration	Not mentioned	

Note 1: ①total effective rate ②IL-6③IL-13④PCT ⑤ENF-α⑥CPIS⑦APACHEⅡ⑧hs-CRP⑨LAC⑩invasive mechanical ventilation⑪transfer rate of ICU.

### 3.3 Meta-analysis

#### 3.3.1 Overall clinical efficacy rate

Eleven studies (Meng, 2016; Li J, and Xue C J, 2018; Tong, 2018; Xu T Y et al., 2018; Zhao XF, 2018; Deng WF, 2019; Gao, 2020; Chengfeng, and Ji, 2021; Yu and Hu, 2021; Li S et al., 2022; Zhang B Z et al., 2023) reported the overall efficacy rate. There was low heterogeneity (P = 0.07, I^2^ = 41%), and a fixed-effects model showed that the experimental group had a higher response rate than controls [RR = 1.21, 95% CI (1.14, 1.28), P < 0.00001].

**FIGURE 4:**
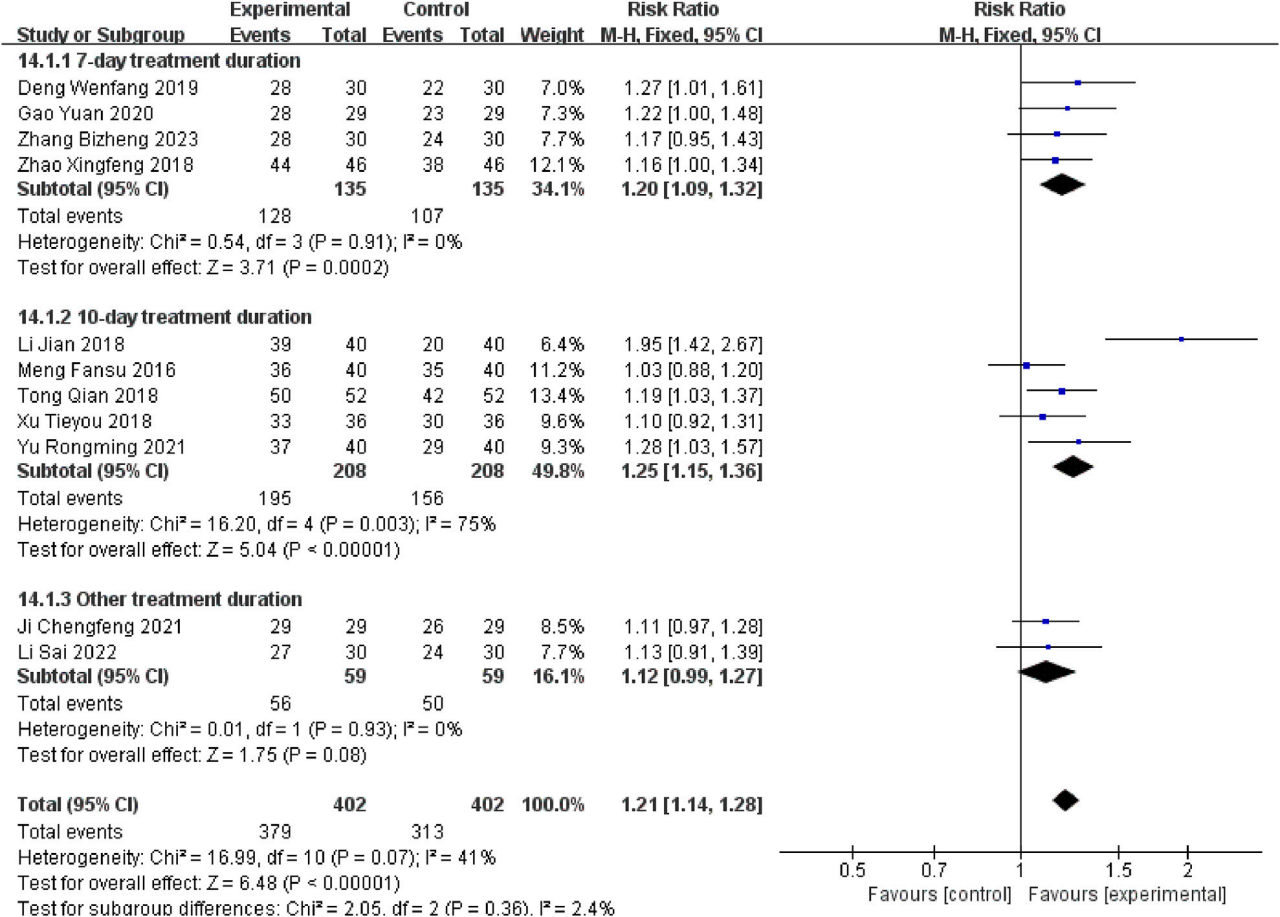
Overall clinical efficacy rate.

Stratified subgroup analyses were conducted according to therapeutic intervention duration. Studies employing a 7-day therapeutic regimen (Zhao XF, 2018; Deng WF, 2019; Gao, 2020; Zhang B Z et al., 2023) demonstrated a measurable therapeutic advantage for the experimental group [RR = 1.20, 95% CI (1.09, 1.32), P = 0.0002]. Compared to this baseline, extended 10-day treatment protocols (Meng, 2016; Li J, and Xue C J, 2018; Tong, 2018; Xu T Y et al., 2018; Yu and Hu, 2021) yielded significantly enhanced outcomes [RR = 1.25, 95% CI (1.15, 1.36), P < 0.00001], with Xuanbai Chengqi Decoction exhibiting superior efficacy in the extended treatment cohort relative to controls. However, heterogeneity testing results (Chi^2^ = 16.20, df = 4, P = 0.003, I^2^ = 75%) revealed significant heterogeneity, possibly due to differences in treatment protocols, patient characteristics, or disease severity. Nevertheless, the overall effect test (Z = 5.04, P < 0.00001) indicates a statistically significant adjunctive effect of the decoction with a 10-day course. In other subgroup studies with different durations (Chengfeng, and Ji, 2021; Li S et al., 2022)[RR = 1.12, 95% CI (0.99, 1.27), P = 0.08], Although the results trended towards favouring the experimental group, the 95% confidence interval encompassed the null value (Figure 4), reflecting no statistically significant difference, which may be attributed to limited sample sizes or variability in treatment durations.

#### 3.3.2 PCT, LaC, hs-CRP, IL-13

**FIGURE 5:**
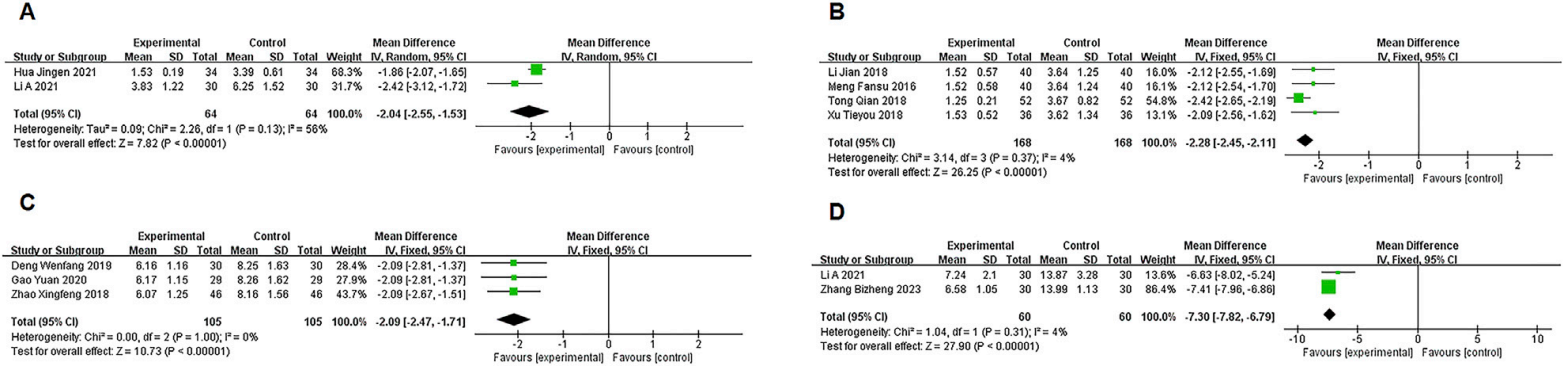
**(A)** PCT, **(B)** LaC, **(C)** hs-CRP, **(D)** IL-13.

Two studies (Hua, 2021; Li E et al., 2021) reported data on PCT, with moderate heterogeneity (P = 0.13, I^2^ = 56%). A random-effects model meta-analysis showed a significant difference between the experimental and control groups in PCT levels [MD = −2.04, 95% CI (−2.55, −1.53), P < 0.00001], favoring the experimental group (Figure 5A).

Four studies (Meng, 2016; Li J, and Xue C J, 2018; Tong, 2018; Xu T Y et al., 2018) provided LaC data, showing no significant heterogeneity (P = 0.34, I^2^ = 4%). A fixed-effects model meta-analysis indicated a significant difference in LaC levels between the groups [MD = −2.28, 95% CI (−2.45, −2.11), P < 0.00001], with the experimental group showing a notable reduction in LaC levels (Figure 5B).

Three studies (Zhao XF, 2018; Deng WF, 2019; Gao, 2020) provided data on hs-CRP, with no significant heterogeneity (P = 1.00, I^2^ = 0%). A fixed-effects model meta-analysis showed a significant difference in hs-CRP levels between the two groups [MD = −2.09, 95% CI (−2.47, −1.71), P < 0.00001], with the experimental group showing marked improvement (Figure 5C).

Two studies (Li E et al., 2021; Zhang B Z et al., 2023) reported IL-13 data, with no significant heterogeneity (P = 0.31, I^2^ = 4%). A fixed-effects model meta-analysis revealed a significant difference in IL-13 levels between the groups [MD = −7.30, 95% CI (−7.82, −6.79), P < 0.00001], indicating a notable advantage for the experimental group (Figure 5D).

#### 3.3.3 IL-6 and TNF-α subgroup analysis

**FIGURE 6:**
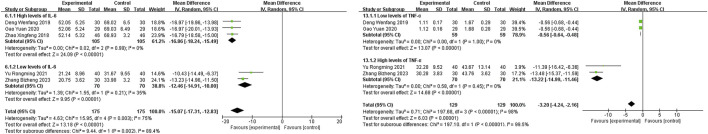
IL-6 and TNF-α subgroup analysis.

In five studies (Zhao XF, 2018; Deng WF, 2019; Gao, 2020; Yu and Hu, 2021; Zhang B Z et al., 2023) reporting IL-6 data, significant heterogeneity was observed (P = 0.003, I^2^ = 75%). A random-effects model was used, with subgroup analysis based on IL-6 levels. In the high IL-6 level subgroup (Zhao XF, 2018; Deng WF, 2019; Gao, 2020), the experimental group showed a significantly lower IL-6 level than the control group [MD = −16.86, 95% CI (−18.24, −15.49), P < 0.00001], demonstrating the efficacy of Xuanbai Chengqi Decoction in reducing IL-6 levels in patients with high IL-6. For the low IL-6 level subgroup (Yu and Hu, 2021; Zhang B Z et al., 2023), the experimental group also showed a significantly lower IL-6 level than the control group [MD = −12.46, 95% CI (−14.91, −10.00), P < 0.00001], though to a slightly lesser degree than the high IL-6 subgroup. Heterogeneity testing between subgroups (Chi^2^ = 9.44, df = 1, P = 0.002, I^2^ = 89.4) indicated significant differences in treatment effects between high and low IL-6 level patients, with greater improvement observed in the high IL-6 subgroup. This may reflect the stronger anti-inflammatory effect of the decoction in patients with higher inflammation levels. Overall, the comparison between the experimental and control groups in IL-6 levels was statistically significant [MD = −15.07, 95% CI (−17.31, −12.83), P < 0.00001], supporting the efficacy of Xuanbai Chengqi Decoction as an adjunct in severe pneumonia treatment (Figure 6A).

“Four studies (Deng WF, 2019; Gao, 2020; Yu and Hu, 2021; Zhang B Z et al., 2023) reported data on TNF-α, with significant heterogeneity (P < 0.00001, I2 = 98%). A random-effects model was used to process the data. Subgroup analysis based on TNF-α count levels showed that the high TNF-α count subgroup Yu and Hu, 2021; Zhang B Z et al., 2023) had a mean difference (MD) of −13.22 [95% CI (−14.99, −11.46), P < 0.00001], significantly lower than the control group. The low TNF-α count subgroup (Deng WF, 2019; Gao, 2020) showed an MD of −0.56 [95% CI (−0.64, −0.48), P < 0.00001], also significantly lower than the control group. Heterogeneity testing between subgroups (Chi2 = 197.10, df = 1, P < 0.00001, I2 = 99.5) suggested significant differences in the effects of Xuanbai Chengqi Decoction treatment between high and low TNF-α level patients. The MD for high TNF-α patients (−13.22) was greater than that for low TNF-α patients (−0.56), indicating that Xuanbai Chengqi Decoction had a more significant improvement effect on patients with high TNF-α levels. However, the overall results showed that the difference in TNF-α between the experimental and control groups was statistically significant [MD = −3.20, 95% CI (−4.24, −2.16), P < 0.00001], indicating that Xuanbai Chengqi Decoction has a significant overall effect in the adjunctive treatment of severe pneumonia (Figure 6B).

Whilst the present meta-analysis suggested a potential therapeutic effect of Xuanbai Chengqi Decoction on IL-6 and TNF-α level reduction, substantial heterogeneity persisted across both primary and subgroup analyses, likely attributable to methodological disparities in trial design, heterogeneous patient demographics, and variations in treatment protocols, including dosing regimens and intervention durations. Furthermore, since IL-6 and TNF-α quantification requires clinical context and specific case details, variations between laboratories and studies may exist. Thus, the specific values and grading of IL-6 and TNF-α remain debated, and subgroup analysis based on this plan carries some subjectivity. Therefore, future studies should consider increasing sample size, standardizing efficacy evaluation criteria, and further exploring the stratified role of IL-6 and TNF-α levels in severe pneumonia patients to enhance the reliability and applicability of the results.

#### 3.3.4 APACHE II

**FIGURE 7:**
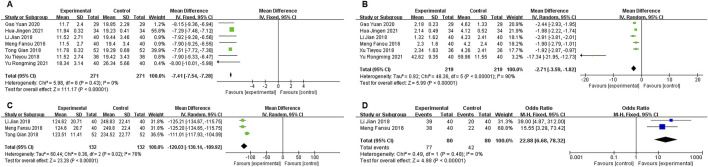
**(A)** APACHE II, **(B)** CPIS, **(C)** Mechanical ventilation duration, **(D)** ICU transfer rate.

Seven studies (Meng, 2016; Li J, and Xue C J, 2018; Tong, 2018; Xu T Y et al., 2018; Gao, 2020; Hua, 2021; Yu and Hu, 2021) reported data on APACHE II scores, with no significant heterogeneity (P = 0.43, I2 = 0%). A fixed-effects model was used for the meta-analysis, showing a statistically significant difference in APACHE II scores between the two groups [MD = −7.41, 95% CI (−7.54, −7.28), P < 0.00001], with the experimental group showing a significant reduction in APACHE II scores (Figure 7A).

#### 3.3.5 CPIS

Six studies (Meng, 2016; Li J, and Xue C J, 2018; Tong, 2018; Xu T Y et al., 2018; Gao, 2020; Hua, 2021; Yu and Hu, 2021) reported data on CPIS scores, with significant heterogeneity (P < 0.00001, I2 = 90%). A random-effects model was used for the meta-analysis, showing a statistically significant difference in CPIS scores between the two groups [MD = −2.71, 95% CI (−3.95, −1.82), P < 0.00001], with the experimental group showing a significant reduction in CPIS scores (Figure 7B).

#### 3.3.6 Mechanical ventilation duration

Three studies (Meng, 2016; Li J, and Xue C J, 2018; Tong, 2018) reported data on mechanical ventilation duration, with significant heterogeneity (P < 0.00001, I2 = 76%). A random-effects model was used for the meta-analysis, showing a statistically significant difference in mechanical ventilation duration between the two groups [MD = −120.03, 95% CI (−130.14, −109.92), P < 0.00001], with the experimental group showing a significant reduction in mechanical ventilation duration (Figure 7C).

#### 3.3.7 ICU transfer rate

Two studies (Meng, 2016; Li J, and Xue C J, 2018) reported data on ICU transfer rates, with significant heterogeneity (P < 0.00001, I2 = 0%). A fixed-effects model was used for the meta-analysis, showing a statistically significant difference in ICU transfer rates between the two groups [22.88, 95% CI (6.68, 78.32), P < 0.00001], with the experimental group showing a significant reduction in ICU transfer rates (Figure 7D).

#### 3.3.8 Adverse reactions

Of the 14 included studies, two (Meng, 2016; Xu T Y et al., 2018) observed the safety of the drug, and no significant adverse reactions were observed in either the experimental or control group. The remaining studies did not report safety assessment methodologies, potentially underestimating pharmacological risks. Future investigations should prioritise systematic documentation of adverse events to enable comprehensive safety profiling.

#### 3.3.9 Sensitivity analysis

**FIGURE 8:**
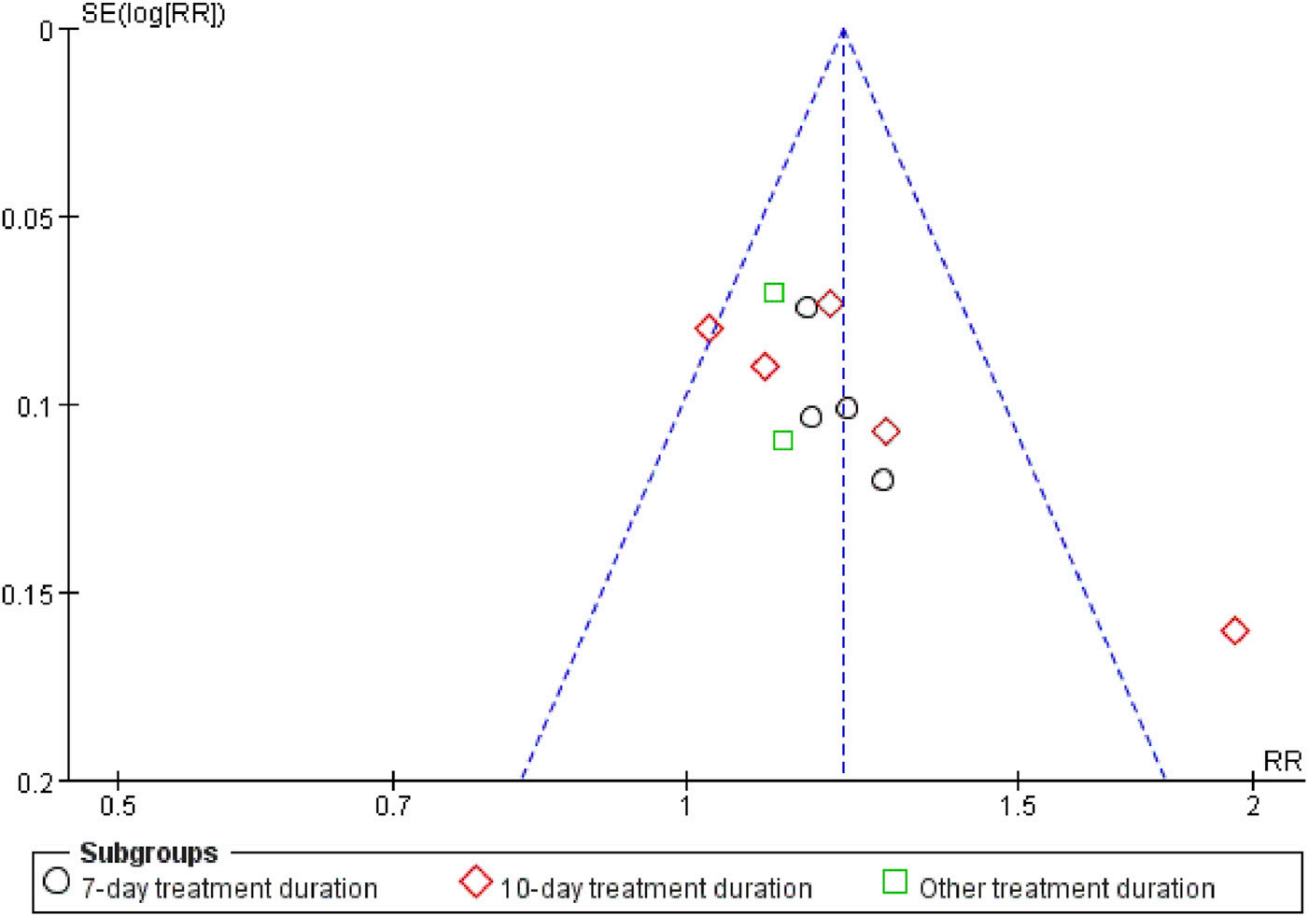
Sensitivity analysis.

Sensitivity analyses of outcomes with at least two trials showed stable findings for IL-6, IL-13, TNF-α, hs-CRP, LaC, APACHE II, and ICU transfer rates. However, effect sizes for PCT, mechanical ventilation duration, and CPIS were more sensitive, suggesting less robust results. A funnel plot assessing publication bias for overall clinical efficacy was asymmetrical, implying a possibility of publication bias related to selective reporting or limited study quality (Figure 8).

## 4 Discussion

Severe pneumonia (SP), an acute pulmonary infection induced by pathogenic microorganisms, still leads to elevated incidence and mortality, especially with an aging population, a higher prevalence of immunocompromised states, evolving pathogens, and the surge in antibiotic resistance (Shorr and Zilberberg, 2021; Vadász et al., 2021). In traditional Chinese medicine (TCM), severe pneumonia is classified as a “cough” disorder under the syndrome pattern of phlegm-heat obstructing lung (tan re zu fei). This condition arises from the interaction of exogenous pathogens transforming into heat and retained phlegm-dampness, leading to stagnation of the lung collaterals. Clinical manifestations include productive cough with dyspnoea, yellow viscous sputum, chest pain, fever, red tongue with yellow greasy coating, and slippery rapid pulse.

Diagnosis followed the criteria outlined in *Chinese Internal Medicine* (Zhong Yi Nei Ke Xue), integrating symptomology, physical signs, and tongue/pulse characteristics. All included studies explicitly adopted this syndrome pattern as the therapeutic indication (Table 2), ensuring that the meta-analysis findings are applicable to severe pneumonia (SP) patients meeting these TCM diagnostic criteria. In current clinical practice, serum biomarkers serve as essential tools for prognosticating infectious diseases (Julkunen et al., 2021). Among these, procalcitonin (PCT) and C-reactive protein (CRP) have been widely researched and linked to poorer outcomes (Florin et al., 2020; Gautam et al., 2020). Cytokines such as IL-6, IL-13, and TNF-α have also been associated with disease severity and mortality (Qu et al., 2020). Standard treatment emphasizes broad-spectrum antibiotics, mechanical ventilation, and steroid therapy, although their use is constrained by drug resistance and adverse effects, fueling the quest for alternative treatments.

Xuanbai Chengqi Decoction (XBCD) was initially documented in “Wenbing Tiaobian, Zhongjiao Chapter.” According to TCM theory, pneumonia develops when excessive phlegm-heat obstructs lung qi flow. XBCD integrates ingredients such as gypsum (Shi Gao), rhubarb (Da Huang), bitter apricot seed (Xing Ren), and Snakegourd Peel (Gua Lou Pi). Gypsum reduces heat, rhubarb dispels heat through its purgative effect, and bitter apricot seed with snakegourd peel moisten the lungs and dissolve phlegm (Wang et al., 2023; Wang et al., 2024). Modern pharmacological research indicates that XBCD can downregulate inflammatory mediators, notably IL-6 and TNF-α, thereby mitigating lung inflammation. It also potentially modulates immune responses to pathogens (Luo et al., 2024; Yichuan et al., 2024). In the progression of severe pneumonia, rhubarb can significantly protect the intestinal barrier, promote the repair of AT2 cells, and inhibit the accumulation of CD11bLy6G + variable aberrant neutrophils by regulating the S100A8 protein. Simultaneously, rhubarb significantly reduced intestinal inflammation and barrier damage by regulating IL-6, IL-1β, and tight junction protein levels (Claudin-1 and ZO-1), improving intestinal barrier permeability (Guo et al., 2024). The calcium sulphate in gypsum mitigates acute inflammatory responses through suppression of the NF-κB pathway, thereby reducing the release of pro-inflammatory cytokines, including IL-6 and TNF-α, alongside modulation of oxidative stress and calcium ion channel activity (Huo et al., 2022). Amygdalin, the bioactive compound in bitter apricot seeds, inhibits Toll-like receptor 4 (TLR4) signalling, attenuating neutrophil infiltration and inflammasome activation, while concurrently restoring immune homeostasis via regulation of the T helper 17 (Th17)/regulatory T cell (Treg) balance. Trichosanthin and flavonoid constituents isolated from snakegourd peel demonstrate cyclooxygenase-2 (COX-2) inhibitory activity, leading to diminished prostaglandin E2 (PGE2) synthesis, reduced vascular permeability, and suppression of inflammatory exudation. These effects are mechanistically linked to peroxisome proliferator-activated receptor gamma (PPAR-γ) pathway activation, which impedes NOD-like receptor protein 3 (NLRP3) inflammasome assembly and subsequent interleukin-1 beta (IL-1β) maturation and secretion (Wang et al., 2024). Furthermore, polysaccharides derived from snakegourd peel induce polarisation of alveolar macrophages towards the M2 anti-inflammatory phenotype, enhancing phagocytic function and promoting inflammatory resolution. Collectively, these pharmacological actions ameliorate pulmonary inflammatory exudation and airway hyperresponsiveness, providing mechanistic evidence for the clinical efficacy of Modified Xuanbai Chengqi Decoction in reducing mechanical ventilation duration and improving CPIS outcomes (Huo et al., 2022; Luo et al., 2024).

In this systematic review and meta-analysis, we found that Modified XBCD notably lowered IL-6, IL-13, and TNF-α, improved clinical scoring systems (APACHE II, CPIS), shortened mechanical ventilation, and boosted ICU discharge rates. Subgroup analyses on IL-6 and TNF-α further indicated more pronounced benefits for patients with elevated baseline cytokine levels, suggesting that XBCD could assist in managing systemic inflammation, particularly in cases characterized by a cytokine storm. A 7–10 days course appeared most favorable in enhancing clinical endpoints, and no significant adverse events were reported. Thus, Modified XBCD emerges as a potentially safe adjunct for individuals resistant or intolerant to conventional Western approaches, offering an alternative for multidrug-resistant infections.

Nevertheless, the study has several limitations. The majority of included trials were from China, with concerns about their methodological quality and possible biases. Heterogeneity was considerable, likely due to variability in patient severity, treatment regimens, and observation periods. Most investigations emphasized inflammatory markers, while long-term survival and quality-of-life data remain scarce. Rigorous, high-quality RCTs are essential to confirm these findings, clarify the safety profile, and expand the therapeutic scope for severe pneumonia.

## 5 Conclusion

Modified Xuanbai Chengqi Decoction appears to exert significant effects in treating severe pneumonia. It may serve as a viable component of integrative therapy to enhance clinical outcomes. Notwithstanding the clinical applicability concerns arising from limited safety evidence—where merely 14.3% of studies reported adverse reactions alongside methodological constraints exacerbated by inadequate sample sizes—these limitations collectively underscore the imperative for rigorously designed, large-scale trials to validate current findings and enable evidence-based integration of traditional Chinese medicine in severe pneumonia management.

## Data Availability

The original contributions presented in the study are included in the article/Supplementary Material, further inquiries can be directed to the corresponding author.

## References

[B1] Bergmann F. Radtke C. Zeitlinger M. Jorda A. (2024). Revisiting the evidence: corticosteroid efficacy in patients with moderate and severe community-acquired pneumonia. Clin. Infect. Dis. 78 (6), 1775. 10.1093/cid/ciad765 38170869

[B2] Cheng L. Yuan-Hang Ye Guo-Guang S. Xiao-dong Li Lan Z. Jia Ke (2023). Research progress of Xuanbai Chengqi Decoction and its additive prescription in the treatment of respiratory diseases. J. Propr. Chin. Med. 45 (10), 3369–3375.

[B3] Chengfeng Ji Ji H. (2021). Clinical observation of Xuanbai Chengqi Decoction in treating severe pneumonia with phlegm heat obstructing lung type %J Journal of Practical Chinese Medicine. 37(06), 1032–1033.

[B4] Cillóniz C. Torres A. Niederman M. S. (2021). Management of pneumonia in critically ill patients. Bmj 375, e065871. 10.1136/bmj-2021-065871 34872910

[B5] Decker B. K. Forrester L. A. Henderson D. K. (2022). Management of unique pneumonias seen in the intensive care unit. Infect. Dis. Clin. North Am. 36 (4), 825–837. 10.1016/j.idc.2022.07.003 36328638

[B6] Deng W. F. (2019). Clinical effect of Xuanbai Chengqi Decoction in the treatment of severe pneumonia with phlegm-heat obstructing lung %J Inner Mongolia Traditional. Chin. Med. 38 (08), 44–45. 10.16040/j.cnki.cn15-1101.2019.08.026

[B7] Florin T. A. Ambroggio L. Brokamp C. Zhang Y. Rattan M. Crotty E. (2020). Biomarkers and disease severity in children with community-acquired pneumonia. Pediatrics 145 (6), e20193728. 10.1542/peds.2019-3728 32404432 PMC7263054

[B8] Gao Y. (2020). Observation on the curative effect of Xuanbai Chengqi Decoction on severe pneumonia of sputum heat obstructing lung type %J Chinese Practical Medicine. 15(07), 184–186. 10.14163/j.cnki.11-5547/r.2020.07.081

[B9] Gautam S. Cohen A. J. Stahl Y. Valda Toro P. Young G. M. Datta R. (2020). Severe respiratory viral infection induces procalcitonin in the absence of bacterial pneumonia. Thorax 75 (11), 974–981. 10.1136/thoraxjnl-2020-214896 32826284

[B10] Guo L. Bao W. Yang S. Liu Y. Lyu J. Wang T. (2024). Rhei Radix et Rhizoma in Xuanbai-Chengqi decoction strengthens the intestinal barrier function and promotes lung barrier repair in preventing severe viral pneumonia induced by influenza A virus. J. Ethnopharmacol. 319 (Pt 2), 117231. 10.1016/j.jep.2023.117231 37783404

[B11] Haessler S. Guo N. Deshpande A. Zilberberg M. D. Lagu T. Lindenauer P. K. (2022). Etiology, treatments, and outcomes of patients with severe community-acquired pneumonia in a large U.S. Sample. Crit. Care Med. 50 (7), 1063–1071. 10.1097/ccm.0000000000005498 35191410 PMC9233133

[B12] Hua J. (2021). Analysis of curative effect of Xuanbai Chengqi Decoction in treating severe pneumonia with phlegm heat obstructing lung type %J Journal of Practical Chinese Medicine. 37(03), 419–420.

[B13] Huo J. Wang T. Wei B. Shi X. Yang A. Chen D. (2022). Integrated network pharmacology and intestinal flora analysis to determine the protective effect of Xuanbai-Chengqi decoction on lung and gut injuries in influenza virus-infected mice. J. Ethnopharmacol. 298, 115649. 10.1016/j.jep.2022.115649 35987410

[B14] Julkunen H. Cichońska A. Slagboom P. E. Würtz P. Nightingale Health UK Biobank Initiative (2021). Metabolic biomarker profiling for identification of susceptibility to severe pneumonia and COVID-19 in the general population. Elife 10, e63033. 10.7554/eLife.63033 33942721 PMC8172246

[B15] Li E. Chen R. Jiajie L. V. (2021). Effect of Xuanbai Chengqi Decoction on severe pneumonia with phlegm-heat obstructing lung and its influence on PCT and IL-13 %J Chinese. J. Traditional Chin. Med. 39 (08), 242–244. 10.13193/j.iSSN.1673-7717.2021.08.058 33522193

[B16] Li J. Wang Y. Zhao W. Yang T. Zhang Q. Yang H. (2024). Multi-omics analysis reveals overactive inflammation and dysregulated metabolism in severe community-acquired pneumonia patients. Respir. Res. 25 (1), 45. 10.1186/s12931-024-02669-6 38243232 PMC10797892

[B17] Li J. Xue C. J. (2018). Clinical observation of Xuanbai Chengqi Decoction in the treatment of severe pneumonia with phlegm-heat obstructing lung %J Guangming Chinese Medicine. 33(04), 526–528.

[B18] Li S. Zhang L. F. Zheng L. Z. (2022). Clinical observation of xuanbaicheng qi-decoction in the treatment of severe pneumonia %J journal of practical Chinese medicine. 38(10), 1741–1743.

[B19] Li Y. Shen D. Wang K. Xue Y. Liu J. Li S. (2023). Mogroside V ameliorates broiler pulmonary inflammation via modulating lung microbiota and rectifying Th17/Treg dysregulation in lipopolysaccharides-induced lung injury. Poult. Sci. 102 (12), 103138. 10.1016/j.psj.2023.103138 37862871 PMC10590742

[B20] Liu Y. N. Zhang Y. F. Xu Q. Qiu Y. Lu Q. B. Wang T. (2023). Infection and co-infection patterns of community-acquired pneumonia in patients of different ages in China from 2009 to 2020: a national surveillance study. Lancet Microbe 4 (5), e330–e339. 10.1016/s2666-5247(23)00031-9 37001538 PMC12514336

[B21] Luo C. Ye Y. H. Jiang C. Ning B. Ke J. Chen G. (2024). Mechanism of Xuanbai Chengqi Decoction in treating acute lung injury based on network pharmacology and experimental verification. Zhongguo Zhong Yao Za Zhi 49 (16), 4329–4337. 10.19540/j.cnki.cjcmm.20240513.702 39307770

[B22] Meng F. S. U. (2016). Observation on the curative effect of Xuanbai Chengqi Decoction on severe pneumonia of phlegm-heat obstructing lung type %J Hebei Chinese Medicine. 38(01), 92–94.

[B23] Mizgerd J. P. (2017). Pathogenesis of severe pneumonia: advances and knowledge gaps. Curr. Opin. Pulm. Med. 23 (3), 193–197. 10.1097/mcp.0000000000000365 28221171 PMC5492380

[B24] Nair G. B. Niederman M. S. (2021). Updates on community acquired pneumonia management in the ICU. Pharmacol. Ther. 217, 107663. 10.1016/j.pharmthera.2020.107663 32805298 PMC7428725

[B25] Qu X. Xu Z. Lin X. (2020). Effects of different doses of methylprednisolone on TNF-α, IL-6, and IL-13 in serum and bronchoalveolar lavage fluid of children with severe mycoplasma pneumoniae pneumonia. J. Biol. Regul. Homeost. Agents 34 (5), 1889–1895. 10.23812/20-317-l 32996302

[B26] Shorr A. F. Zilberberg M. D. (2021). Novelty and nuance in the intensive care unit: new options to combat multidrug resistant pneumonia. Curr. Opin. Infect. Dis. 34 (2), 151–155. 10.1097/qco.0000000000000712 33395092

[B27] Tong Q. (2018). Clinical observation of combined Chinese and Biomedicine in the treatment of severe pneumonia with phlegm-heat obstructing pulmonary type %J Journal of Practical Chinese Medicine. 34(02), 238–239.

[B28] Vadász I. Husain-Syed F. Dorfmüller P. Roller F. C. Tello K. Hecker M. (2021). Severe organising pneumonia following COVID-19. Thorax 76 (2), 201–204. 10.1136/thoraxjnl-2020-216088 33177230

[B29] wang C.-lin (2015). XuanBaiCheng gas soup clinical observation on 21 patients with mechanical ventilation treatment of severe pneumonia % J zhejiang. J. traditional Chin. Med. 50 (02), 99. 10.13633/j.carol.carrollnki.ZJTCM.2015.02.013

[B30] Wang G. Yuzuo G. Yuanyuan W. Zhu H. X. Jiang Y. (2024). Randomized controlled study of Xuanbai Chengqi Decoction on immune imbalance in children with mycoplasma pneumonia. J Chin. J. Integr. Med. 44 (04), 414–418.

[B31] Wang S. Lin F. Zhang C. Gao D. Qi Z. Wu S. (2024). Xuanbai Chengqi Decoction alleviates acute lung injury by inhibiting NLRP3 inflammasome. J. Ethnopharmacol. 319 (Pt 2), 117227. 10.1016/j.jep.2023.117227 37751794

[B32] Wang Z. Kejing Yu Liu Q. Zhou X. Shen J. (2023). Clinical observation of 30 cases of severe traumatic ventilator-associated pneumonia with phlegm-heat obstructing lung treated with modified Xuanbai Chengqi Decoction. J J. TCM 64 (03), 269–274. 10.13288/J.11-2166/r.2023.03.010

[B33] Xu T. Y. Sun Y. C. Fei X. Y. (2018). Effect analysis of Xuanbai Chengqi Decoction in the treatment of severe pneumonia with phlegm-heat obstruction %J Inner Mongolia Traditional. Chin. Med. 37 (12), 26–27. 10.16040/j.cnki.cn15-1101.2018.12.017

[B34] Yang S. Lu S. Guo Y. Luan W. Liu J. Wang L. (2024). A comparative study of general and severe mycoplasma pneumoniae pneumonia in children. BMC Infect. Dis. 24 (1), 449. 10.1186/s12879-024-09340-x 38671341 PMC11046970

[B35] Yichuan C. Junzu S. Wang X. (2024). A study on the metabolomics of Xuanbai Chengqi Decoction flavored-based on the UPLC-Q-Exactive MS technique in the treatment of pneumonia co-infected mice with influenza virus and Streptococcus pneumoniae %J Chinese. J. Traditional Chin. Med. 42 (7), 76–768. 10.13193/j.issn.1673-7717.2024.07.023

[B36] Yu R. M. Hu A. (2021). Effects of Xuanbai Chengqi decoction on CRP, TNF-α, IgG, IL-6 and TCM syndrome scores in patients with severe pneumonia %J sichuan. Chin. Med. 39 (11), 96–99.

[B37] Zhang B. Z. Ding L. Yuan Y. H. Lu M. (2023). Effects of added flavor of Xuanbai Chengqi Decoction on blood gas indexes, serum cytokines, amyloid A and high mobility group protein levels in patients with severe pneumonia. J J. Integr. Chin. Biomed. 32 (23), 3321–3324.

[B38] Zhao X. F. (2018). Clinical efficacy of Xuanbai Chengqi Decoction combined with conventional Biomedicine in the treatment of severe pneumonia with phlegm-heat obstruction and its influence on the levels of inflammatory factor interleukin-6, hypersensitive C-reactive protein and tumor necrosis factor α. J. Hebei Chin. Med. 40 (04), 558–561.

